# SNRPD1/E/F/G Serve as Potential Prognostic Biomarkers in Lung Adenocarcinoma

**DOI:** 10.3389/fgene.2022.813285

**Published:** 2022-03-03

**Authors:** Gaohua Liu, Fuping Li, Meichun Chen, Yang Luo, Yinhai Dai, Peifeng Hou

**Affiliations:** ^1^ Department of Oncology Medicine, Fujian Medical University Union Hospital, Fuzhou, China; ^2^ Department of Clinical Medicine, Shaanxi University of Chinese Medicine, Xianyang, China; ^3^ Department of Hematology, Fujian Medical University Union Hospital, Fuzhou, China; ^4^ Department of Surgical Oncology Medicine, Second Affiliated Hospital of Shaanxi University of Chinese Medicine, Xianyang, China; ^5^ Fujian Medical University Stem Cell Research Institute, Fuzhou, China; ^6^ Fujian Key Laboratory of Translational Cancer Medicine, Fuzhou, China

**Keywords:** SM proteins, prognostic biomarkers, immune infiltration, target therapy, lung adenocarcinoma

## Abstract

**Objectives:** Sm proteins (SNRPB/D1/D2/D3/E/F/G), involved in pre-mRNA splicing, were previously reported in the tumorigenesis of several cancers. However, their specific role in lung adenocarcinoma (LUAD) remains obscure. Our study aims to feature abnormal expressions and mutations of genes for Sm proteins and assess their potential as therapeutic targets *via* integrated bioinformatics analysis.

**Methods:** In this research, we explored the expression pattern and prognostic worth of genes for Sm proteins in LUAD across TCGA, GEO, UALCAN, Oncomine, Metascape, David 6.8, and Kaplan-Meier Plotter, and confirmed its independent prognostic value *via* univariate and multivariate cox regression analysis. Meanwhile, their expression patterns were validated by RT-qPCR. Gene mutations and co-expression of genes for Sm proteins were analyzed by the cBioPortal database. The PPI network for Sm proteins in LUAD was visualized by the STRING and Cytoscape. The correlations between genes for Sm proteins and immune infiltration were analyzed by using the “GSVA” R package.

**Results:** Sm proteins genes were found upregulated expression in both LUAD tissues and LUAD cell lines. Moreover, highly expressed mRNA levels for Sm proteins were strongly associated with short survival time in LUAD. Genes for Sm proteins were positively connected with the infiltration of Th2 cells, but negatively connected with the infiltration of mast cells, Th1 cells, and NK cells. Importantly, Cox regression analysis showed that high SNRPD1/E/F/G expression were independent risk factors for the overall survival of LUAD.

**Conclusion:** Our study showed that SNRPD1/E/F/G could independently predict the prognostic outcome of LUAD and was correlated with immune infiltration. Also, this report laid the foundation for additional exploration on the potential treatment target’s role of SNRPD1/E/F/G in LUAD.

## Introduction

Lung cancer is the most well-known sort of malignant tumor worldwide and is the major cause of cancer mortality (Sung et al., 2021). Lung adenocarcinoma (LUAD) has been the most common subtype of Non-small cell cancer (NSCLC) (Anonymous, 2015). LUAD is characterized by a lack of early clinical symptoms, a high rate of distant metastasis and drug resistance, which pose serious challenges to clinical treatment. Currently, treatment methods for LUAD mainly include surgical resection, radiotherapy, chemotherapy, immunotherapy, and molecular targeted therapy (Reck and Rabe, 2017). The best treatment for lung cancer is surgical resection in the early stages of lung cancer. However, the early symptoms of the disease are not obvious, easy to be ignored, which leads to a late diagnosis. The treatment of advanced lung adenocarcinoma is limited, and molecular targeted therapy is a promising choice, as well as immunotherapy. However, because of the lack of effective molecular targets, most drugs remain ineffective in the treatment of LUAD patients, of whom the 5-year survival rate is just 15% (Chen et al., 2016). Hence, it is absolutely necessary to recognize effective and dependable biomarkers to determine poor prognoses and direct treatment strategies.

The spliceosome is a ribonucleoprotein (RNP) with a complex ring-shaped structure, which mainly consists of small nuclear ribonucleoproteins (snRNPs), which is involved in splicing the pre rna into mature mRNA. Seven Sm proteins (SNRPB, SNRPD1, SNRPD2, SNRPD3, SNRPE, SNRPF, and SNRPG) and a small eponymous small nuclear RNA (snRNA) compose snRNPs (Chari et al., 2008). Accurate splicing is essential for normal cellular functions like cell proliferation, apoptosis, migration, and invasion. Sm proteins, involved in the formation of anti-Sm antibodies, were significant diagnostic biomarkers in autoimmune diseases, such as systemic lupus erythematosus (SLE). Furthermore, priors works have illustrated that the aberrant expressions of genes for Sm proteins are related to some human cancers, including cervical cancer (Zhu et al., 2020), glioblastoma (Correa et al., 2016), breast cancer (Dai et al., 2021), and hepatocellular carcinoma (Zhan et al., 2020).

Up to now, there have been limited studies investigating the connection between the abnormal expressions of genes for Sm proteins and LUAD. For instance, a report (Liu et al., 2019) showed that SNRPB down-regulation inhibited the growth and metastasis of NSCLC cells *via* RAB26 down-regulation. In the study directed by Valles et al. (2012), SNRPB and SNRPE were proved to be related to poor survival in LUAD patients. However, the precise functional roles of Sm proteins in LUAD are unclear yet. Our study aimed to present abnormal expressions and mutations of genes for Sm proteins and assess their potential as therapeutic targets *via* integrated bioinformatics analysis, which may be helpful to the treatment of LUAD patients.

## Materials and Methods

### Data Source and Flow Chart

**FIGURE 1:**
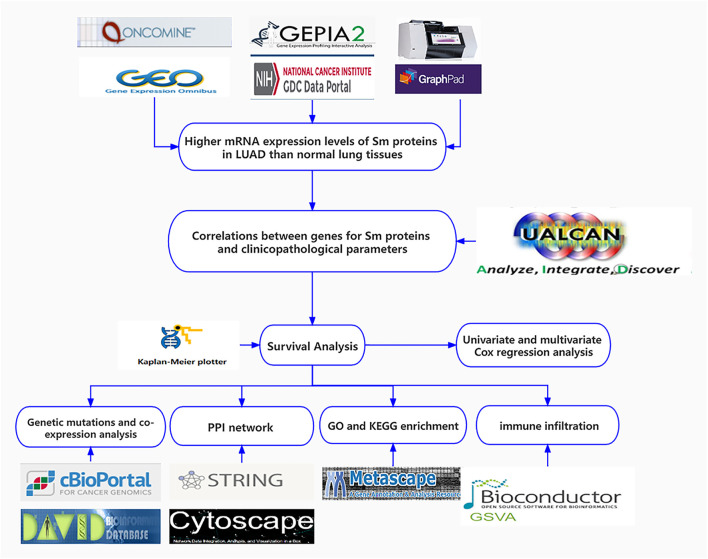
Flow chart of the present study.

The GSE40791 dataset data performed by GPL570 (Zhang et al., 2012) were downloaded from the Gene Expression Omnibus (GEO) database (https://www.ncbi.nlm.nih.gov/geo/) (Clough and Barrett, 2016). GSE40791 included 100 non-tumor lung tissues and 94 lung adenocarcinoma tissues (N = 100, T = 94). The TGCA-LUAD data (N = 59, T = 535) were downloaded from the TGCA database (https://portal.gdc.cancer.gov) (Tomczak et al., 2015). A flow chart of this study procedure is shown in Figure 1. The characteristics of 535 patients, including their ages, genders, smoking history, TNM stages, pathologic stages, primary therapy outcome, residual tumor and anatomic neoplasm subdivision, are presented in Supplementary Table S1.

### Comparison of Genes for Sm Proteins Expression Levels Between LUAD and Corresponding Normal Tissue

The Oncomine database (https://www.oncomine.org) (Rhodes et al., 2004) was used to obtain the data of mRNA expressions for Sm proteins in various cancers, and these data were analyzed *via* Student’s t-test. And we defined the following threshold: *p*-value<0.001, fold change >2 and 10% of most highly ranked genes. Furthermore, we compared expression levels of genes for Sm proteins between lung adenocarcinoma samples and normal samples in GSE40791. Moreover, the transcription levels of these genes were validated in the GEPIA 2 database (http://gepia2.cancer-pku.cn) (Tang et al., 2017), including tumor and normal samples from the TCGA and the Genotype-Tissue Expression Project (GTEx) database (https://gtexportal.org/home/) and the TCGA-LUAD data performed by R software.

### Cell Culture and RT-qPCR

BEAS-2B cell line (human bronchial epithelial cell line) was purchased from the American Type Culture Collection (Manassas, VA, United States), A549 cell line was purchased from the Type Culture Collection of the Chinese Academy of Sciences, Shanghai, China. Two types of cells were cultured in DMEM with 10% FBS, penicillin (50 U/ml), and streptomycin (50 U/ml). The cells were incubated at 37°C with 5% CO_2_.

Total RNA of the above cells was extracted using TRIzol (Thermo, United States), and first-strand complementary DNA (cDNA) synthesis from total RNA was carried out using the GoScript Reverse Transcription System (Promega, United States). RT-qPCR was conducted using an AriaMx Real-Time PCR machine (Agilent Technologies, United States) with TB GreenPremix ExTaq II (Takara Bio, Japan). The primer sequences are shown in Supplementary Table S2. RT-qPCR cycle conditions: 3 min at 95°C, 40 cycles of 15 s for 95°C and 60 s for 60°C. Data was normalized to the house-keeping gene GAPDH. The relative gene expression was performed by the 2^−ΔΔCt^ method (Livak and Schmittgen, 2001).

### Correlations Between Genes for Sm Proteins and Clinicopathological Parameters

In our study, the transcription levels of genes for Sm proteins in TP53 mutation of LUAD patients were analyzed with the lung adenocarcinoma dataset using the UALCAN database (http://ualcan. path.uab.edu) (Chandrashekar et al., 2017). Moreover, we explored the correlation between genes for Sm proteins gene expressions and tumor stages *via* the “Expression DIY module” of GEPIA 2 and selected 50 neighboring genes related to genes for Sm proteins using the “Similar Genes Detection” module.

### Survival Analysis

The Kaplan-Meier plotter (http://kmplot.com) (Gyorffy et al., 2013; Nagy et al., 2021), including mRNA expression data and patients’ clinical information from GEO and TCGA, is a powerful online tool to further verify the prognostic value of genes in several cancers. The overall survival (OS), free progression (FP), and post-progression survival (PPS) curves of genes for Sm proteins in LUAD were shown *via* the Kaplan-Meier plotter. Meanwhile, we also explored if the dyregulation of genes for Sm proteins had any impacts on the OS of LUAD patients with smoke history by using the Kaplan Meier plotter. In addition, univariate and multivariate cox regression analysis of the TGCA-LUAD data was performed using the “survival” and the “survminer” R package.

### Gene Mutations and Co-Expression Analysis

In this study, the lung adenocarcinoma dataset (TCGA, Firehose Legacy), including data from 230 complete samples of 586 patients, was selected and visualized as the map of gene mutations, expression heatmap, and co-expression map of genes for Sm proteins using the cBioPortal database (http://www. cbioportal.org) (Cerami et al., 2012; Gao et al., 2013). The z-Score threshold was set to ±1.8.

### Constructed Protein-Protein Interaction Network and Selected Hub Genes

The tool of STRING (https://string-db.org/) (Szklarczyk et al., 2019) was used to construct the protein-protein interaction (PPI) network between the seven genes for Sm proteins and their 50 frequently neighboring genes. All PPI pairs with a combined score of >0.4 were extracted. Then, we used the “CytoHubba” plugin (v0.1) (Chin et al., 2014) of Cytoscape (v3.8.2) (https://cytoscape.org) (Otasek et al., 2019) to identify hub genes in that PPI network. Furthermore, the online tool of Metascape (http://metascape.org) (Zhou et al., 2019) was used to analyze the MCODE components of that PPI network.

### GO and KEGG Enrichment Analyses

Functional enrichment analyses, including gene ontology (GO) analysis comprising cellular component (CC), molecular function (MF), and biological process (BP), and Kyoto encyclopedia of genes and genomes (KEGG) pathway analysis, were performed *via* the tool of DAVID 6.8 (https://david.ncifcrf.gov) (Huang da et al., 2009). Subsequently, the specific enriched terms for GO and KEGG enrichment analysis were visualized using the “clusterProfiler” package (Yu et al., 2012) in R software. Moreover, we presented the network of enriched terms colored by cluster ID using the online tool of Metascape.

### Associations Between Genes for Sm Proteins and Immune Infiltration

Finally, we explore the associations between genes for Sm proteins and 24 immune cell types in TGCA-LUAD. We investigated dendritic cell (DC), activated dendritic cell (aDC), immature dendritic cell (iDC),plasmacytoid dendritic cell (pDC), B cells, CD8^+^ T cells, Cytotoxic T cells, T cells, T helper cells, T help 1 (Th1) cells, Th17 cells, Th2 cells, T central memory (Tcm), T effector memory (Tem), T follicular helper (Tfh), T gamma delta (Tgd), regulatory T Cell (Treg), eosinophils, macrophages, mast cells, neutrophils, natural killer (NK) cells, NK CD56^bright^ cells and NK CD56^dim^ cells (Bindea et al., 2013), and the above associations was performed using single-sample Gene Sets Enrichment Analysis (ssGSEA) algorithm of R package “GSVA” (Hanzelmann et al., 2013) and lollipop charts were produced using R package “ggplot2.”

### Statistical Analysis

One-way Analysis of Variance (ANOVA) in the GEPIA 2 database, the log-rank test in Kaplan-Meier survival analysis, and the Cox proportional risk models in univariate and multivariate analyses, were used in statistical analysis. R software (v4.0.2) (http://www.r-project.org) was used for analysis in this study. RT-qPCR data was performed in GraphPad Prism (v9.0.2) (San Diego, CA, United States) and presented as the mean ± S.D. Student’s T-test was used for statistical analyses between the data pairs where appropriate. *p < 0.05, **p < 0.01, ***p < 0.001, and ****p < 0.0001 were considered to represent statistical significance.

## Results

### The mRNA Levels of Sm Proteins Were Significantly Increased in Lung Cancer Tissues and LUAD Cell Lines

**FIGURE 2:**
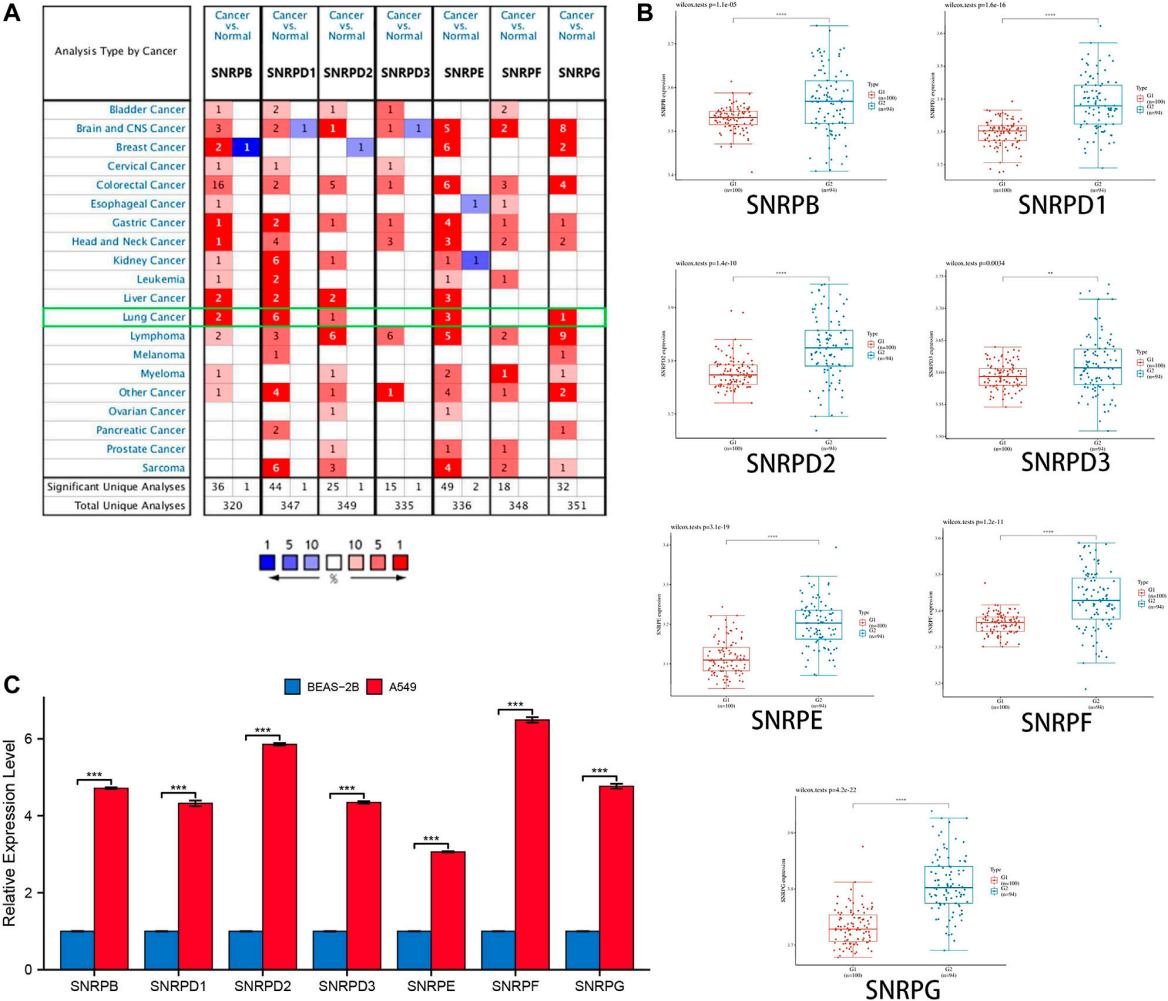
The transcription levels of genes for Sm proteins in lung cancer tissues and lung cell lines. **(A)** The genes for Sm proteins in different human cancers (ONCOMINE). **(B)** Box plots of genes for Sm proteins expression between lung cancer and normal tissues in GSE40791. **(C)** Bar charts of mRNA level of genes for Sm proteins between BEAS-2B and A549 cell line. Students T-test was performed to assess the statistical significance. A p value of <0.05 was regarded as statistically significant.

**FIGURE 3:**
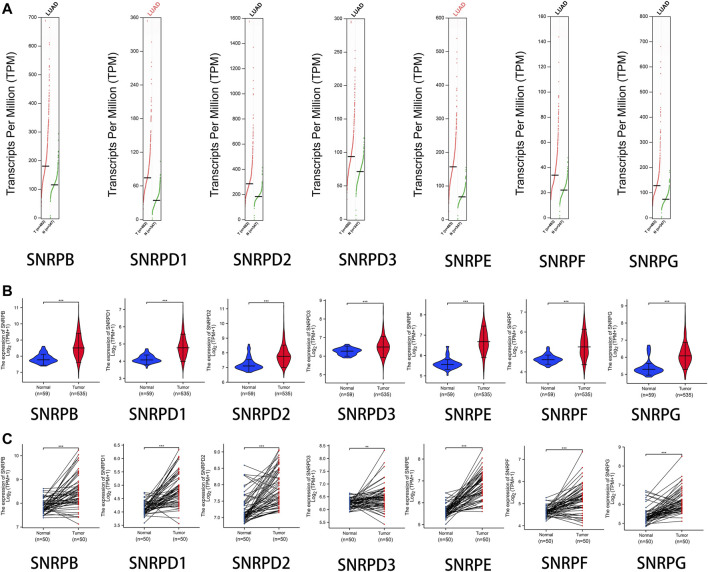
The transcription levels of genes for Sm proteins in LUAD samples and normal lung samples. **(A)** The transcription levels of genes for Sm proteins in data of GEPIA 2 (TCGA-LUAD & GTEx). **(B)** Violin charts of mRNA level for Sm proteins in data of TCGA-LUAD. **(C)** The mRNA expressions of genes for Sm proteins in pair data of TCGA-LUAD. Students T-test was performed to assess the statistical significance. A p value of < 0.05 was regarded as statistically significant.

From Figure 2A and Table 1, we could see that the mRNA levels of SNRPB/D1/D2/E/G were higher in lung cancer than in non-cancer tissues. The results were also consistent with the mRNA levels of Sm proteins were upregulated in LUAD compared to normal lung tissues (Figure 2B). Moreover, the relative mRNA level of all Sm proteins in A549 cell line is higher than the relative mRNA level of them in normal lung cell lines, BEAS-2B (Figure 2C). The transcription levels of genes for Sm proteins considerably increased in data of GEPIA 2 (TCGA-LUAD & GTEx), TCGA-LUAD data and pair LUAD data (Figures 3A–C) match the above results.

**TABLE 1:** The mRNA levels of Sm proteins were significantly higher in lung cancer than in normal lung tissues (ONCOMINE).

	Lung cancer VS normal lung tissue	Fold change	*p*-value	T-test	References
SNRPB	Small cell lung carcinoma	3.338	1.21E-4	6.278	Garber et al. (2001)
	Squamous cell lung carcinoma	2.621	1.52E-4	5.135	Garber et al. (2001)
SNRPD1	Large cell lung carcinoma	2.357	9.91E-5	7.079	Garber et al. (2001)
	Small cell lung carcinoma	2.223	1.40E-4	6.131	Garber et al. (2001)
	Squamous cell lung carcinoma	3.502	5.49E-4	7.549	Yamagata et al. (2003)
	Squamous cell lung carcinoma	2.194	7.89E-17	13.403	Hou et al. (2010)
	Large cell lung carcinoma	2.526	5.01E-8	8.052	Hou et al. (2010)
	Squamous cell lung carcinoma	2.478	9.30E-4	5.237	Wachi et al. (2005)
SNRPD2	Squamous cell lung carcinoma	2.046	8.33E-4	5.359	Wachi et al. (2005)
SNRPD3	NA	NA	NA	NA	NA
SNRPE	Large cell lung carcinoma	2.347	7.58E-9	9.113	Hou et al. (2010)
	Squamous cell lung carcinoma	2.146	2.53E-4	7.002	Wachi et al. (2005)
	Lung carcinoid	2.329	2.86E-4	3.974	Bhattacharjee et al. (2001)
SNRPF	NA	NA	NA	NA	NA
SNRPG	Squamous cell lung carcinoma	2.176	1.80E-5	8.278	Wachi et al. (2005)

NA: not available.

### The Expressions of Genes for Sm Proteins Were Related to Clinicopathological Parameters of LUAD Patients

**FIGURE 4:**
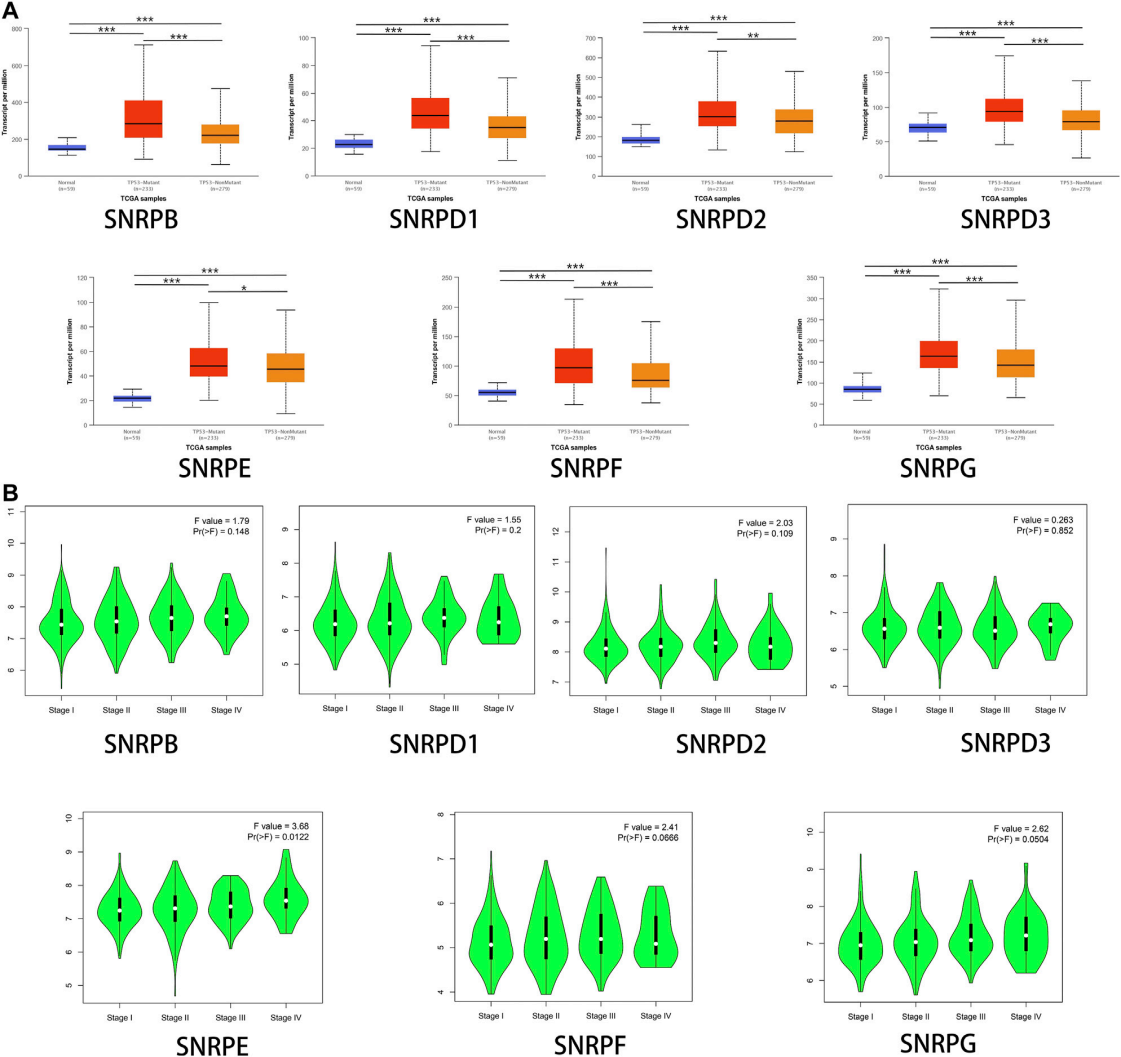
The associations between genes for Sm proteins expression levels and clinicopathological parameters of LUAD patients. **(A)** Correlations between genes for Sm proteins expression levels and TP53 mutation in LUAD (UALCAN). **(B)** Association between mRNA expression of genes for Sm proteins and different tumor stages (GEPIA 2). ANOVA was performed to assess the statistical significance of the variations. F-value indicates the statistical value of the F test; Pr (>F) indicates p value. A p value of <0.05 was regarded as statistically significant.

We research the correlations between the expressions of genes for Sm proteins, TP53 mutation and cancer stages *via* the UALCAN database and the GEPIA2 database. From Figure 4A, we could find that the mRNA expressions of genes for Sm proteins in LUAD with TP53 mutation were higher than those without TP53 mutation (Figure 4A). Moreover, as can be seen in Figure 4B, the mRNA levels of SNRPE varied significantly across different LUAD tumor stages (F value = 3.68, Pr (>F)<0.05), whereas SNRPB/D1/D2/D3/F/G had no significant association with LUAD stages (all Pr (>F) > 0.05).

### The Upregulation of Genes for Sm Proteins is Related to Poor Survival Outcomes in LUAD Patients

**FIGURE 5:**
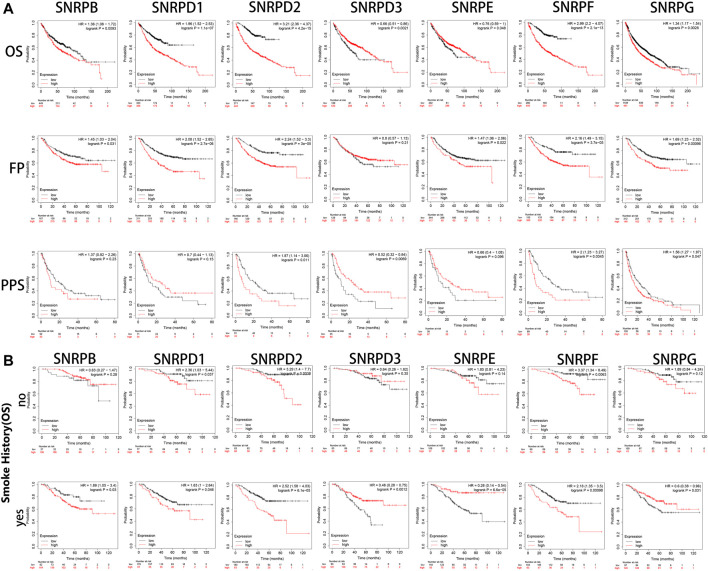
The survival curves of LUAD patients with genes for Sm proteins and impacts of smoking on the survival of these patients (Kaplan-Meier Plotter). **(A)** The Kaplan-Meier survival curves were used to evaluate the survival of LUAD patients with Sm proteins. **(B)** The effects of smoking in the OS of LUAD patients with Sm proteins. The log-rank test was performed in Kaplan-Meier survival analysis. A p value of <0.05 was regarded as statistically significant. HR: hazard ratio.

Using Kaplan-Meier Plotter, we assessed the prognostic values of genes for Sm proteins in LUAD patients. From Figure 5A, we could find out high expressions of genes for Sm proteins were associated with short OS (all *p* < 0.05). In addition, high mRNA level of SNRPB/D1/D2/F (HR > 1, *p* < 0.05) were correlated with poor FP and PPS (all *p* < 0.05). However, highly expressed SNRPD3 was not obviously related to the FP of LUAD patients (*p* > 0.05).

As is well known, smoking is a common risk factor leading to lung cancer (Jassem et al., 2009). Hence, we explored if the upregulation of genes for Sm proteins had any impacts on the overall survival of LUAD patients. From Figure 5B, in the smoking group, the high expression of SNRPB significantly reduced the OS of LUAD patients. However, in the non-smoking group, the expression of SNRPB had no statistical significance for the overall survival of patients with lung adenocarcinoma. This indicates that SNRPB and smoking factors together could promote the development of lung adenocarcinoma. However, the upregulation of SNRPD1 (HR: 2.36, *p* < 0.05 vs HR: 1.63, *p* < 0.05), SNRPD2 (HR: 3.29, *p* < 0.05 vs HR: 2.52, *p* < 0.05), and SNRPF (HR: 3.27, *p* < 0.05 vs HR: 2.18, *p* < 0.05) had worsening impacts on the OS of LUAD patients without smoking history. Noteworthy, only high expression of SNRPE (HR: 1.28, *p* < 0.05) was correlated to poor OS of lung squamous cell carcinoma (LUSC) (Supplementary Figure S1). More research is still needed to interpret potential mechanisms of the above-described events.

Moreover, we attempted to evaluate the independent prognostic values of genes for Sm proteins for OS of LUAD patients. The microarray data and clinical information (Supplementary Table S1) of 535 LUAD patients were obtained from TCGA for Cox regression analysis. Univariate Cox regression analysis demonstrated that T, N, M stage, pathologic stage, SNRPD2, SNRPE, and SNRPF were highly correlated with the short OS of LUAD patients (all *p* < 0.05; Supplementary Table S2). In multivariate Cox regression analysis, SNRPD1, SNRPE, SNRPF, and SNRPG were observed to have significant associations with poor OS of LUAD patients (all *p* < 0.05; Supplementary Figure S2). Taken together, SNRPD1/E/F/G could be considered as independent prognostic factors for poor OS in LUAD.

### Gene Mutations and Co-Expression Analysis of Genes for Sm Proteins in LUAD Patients

**FIGURE 6:**
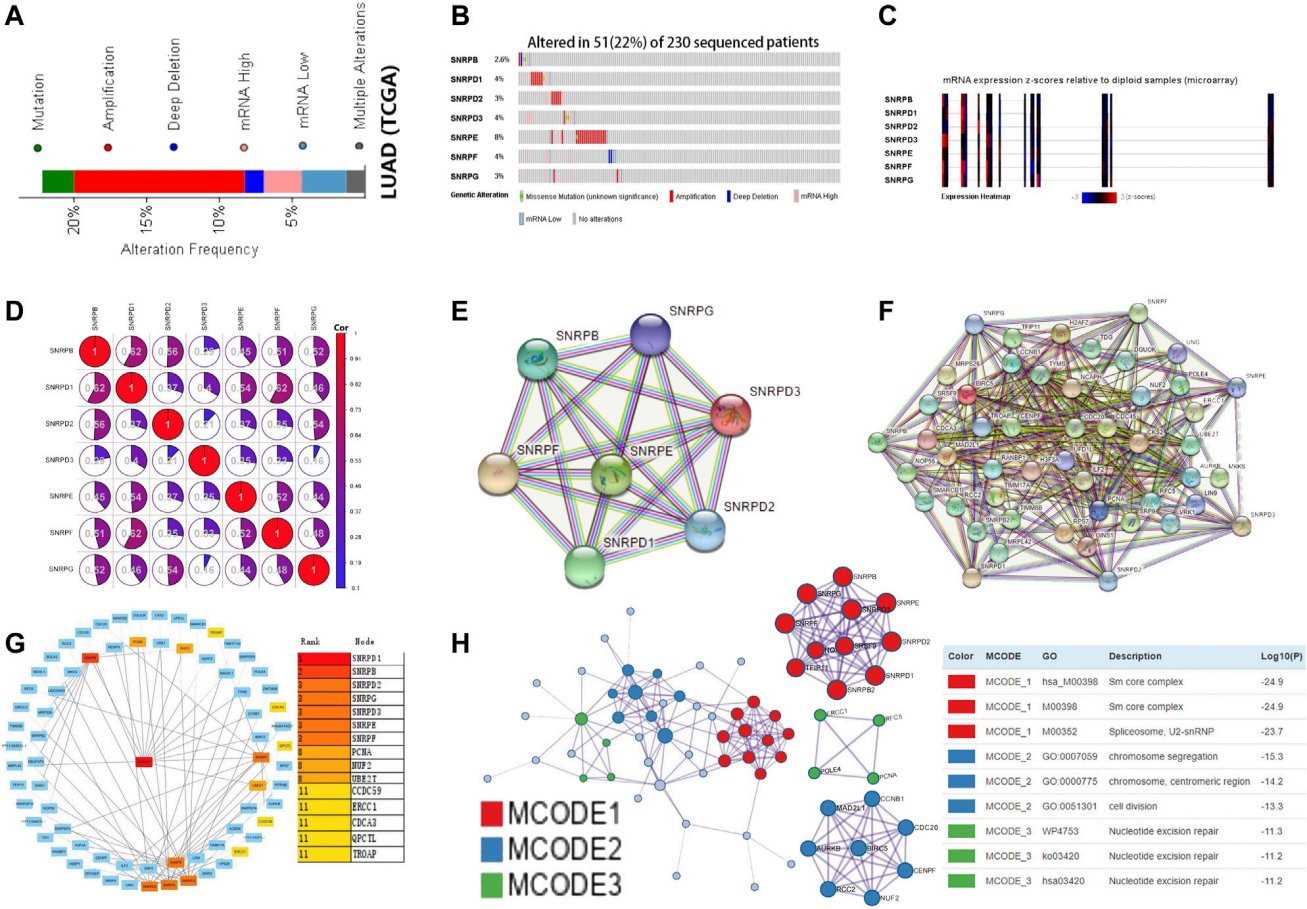
Gene mutations, co-expression, and PPI network for genes for Sm proteins in LUAD (cBioPortal, STRING, Cytoscape, Metascape). **(A–C)** Gene mutations and expression heatmap of genes for Sm proteins in LUAD patients (cBioPortal). **(D)** The co-expression network for genes for Sm proteins (cBioPortal). **(E)** PPI network based on the seven genes for Sm proteins (STRING). **(F)** PPI network based on genes for Sm proteins and their 50 neighbouring proteins (STRING). **(G)** Eight hub genes, including PCNA, NUF2, UBE2T, CCDC59, ERCC1, CDCA3, QPCTC, and TROAP, are shown in the PPI network (Cytoscape). **(H)** Protein-protein interaction network and MCODE components identified in different expressed genes for Sm proteins and 50 most frequently altered neighboring genes (Metascape).

Using the cBioPortal database, we explored the mutation rate and co-expression of Sm proteins. As shown in Figures 6A–C, gene amplification was the most common type of genes for Sm proteins mutations (Figure 6A). 22% (51/230) of LUAD patients were found to have more than one gene mutation. SNRPB, SNRPD1, SNRPD2, SNRPD3, SNRPE, SNRPF, and SNRPG were altered in 2.6%, 4%, 3%, 4%, 8%, 4%, and 3% of the 230 LUAD patients (Figure 6B). Likewise, the Expression Heatmap also displayed the degree of genes for Sm proteins mutations (Figure 6C). SNRPE had the highest mutation rate among genes for Sm proteins in LUAD. Meantime, we performed co-expression analysis for Sm proteins. Significant and positive correlations were observed among genes for Sm proteins in Figure 6D, including SNRPB with SNRPD1/D2/E/F/G, SNRPD1 with SNRPB/D2/D3/E/F/G, SNRPD2 with SNRPB/D1/E/F/G, SNRPD3 with SNRPD1/E/F/G, SNRPE with SNRPB/D1/D2/D3/F/G, SNRPF with SNRPB/D1/D2/D3/E/G, and SNRPG with SNRPB/D1/D2/E/F (Figure 6D).

### Constructed PPI Network and Selected Hub Genes

To explore the possible genes for Sm proteins protein-mediated biological pathways in lung adenocarcinoma, we constructed a protein interaction network based on the seven Sm proteins (Figure 6E) and their 50 frequently neighboring proteins (Figure 6F) *via* the STRING database. Meantime, we used Cytoscape software to find out these hub genes related to genes for Sm proteins-mediated biological pathways. As was shown in Figure 6G, eight hub genes, including PCNA, NUF2, UBE2T, CCDC59, ERCC1, CDCA3, QPCTC, and TROAP, were tightly correlated with the alterations of genes for Sm proteins. Furthermore, we extracted the three most meaningful MCODE components from the PPI network between Sm proteins and their 50 frequently neighboring proteins. As was shown in Figure 6H, Sm core complex, spliceosome, U2-SnRNP, chromosome segregation, chromosome, centromeric region, cell division, and nucleotide excision repair were related to biological function.

### GO and KEGG Enrichment Analyses in LUAD Patients

**FIGURE 7:**
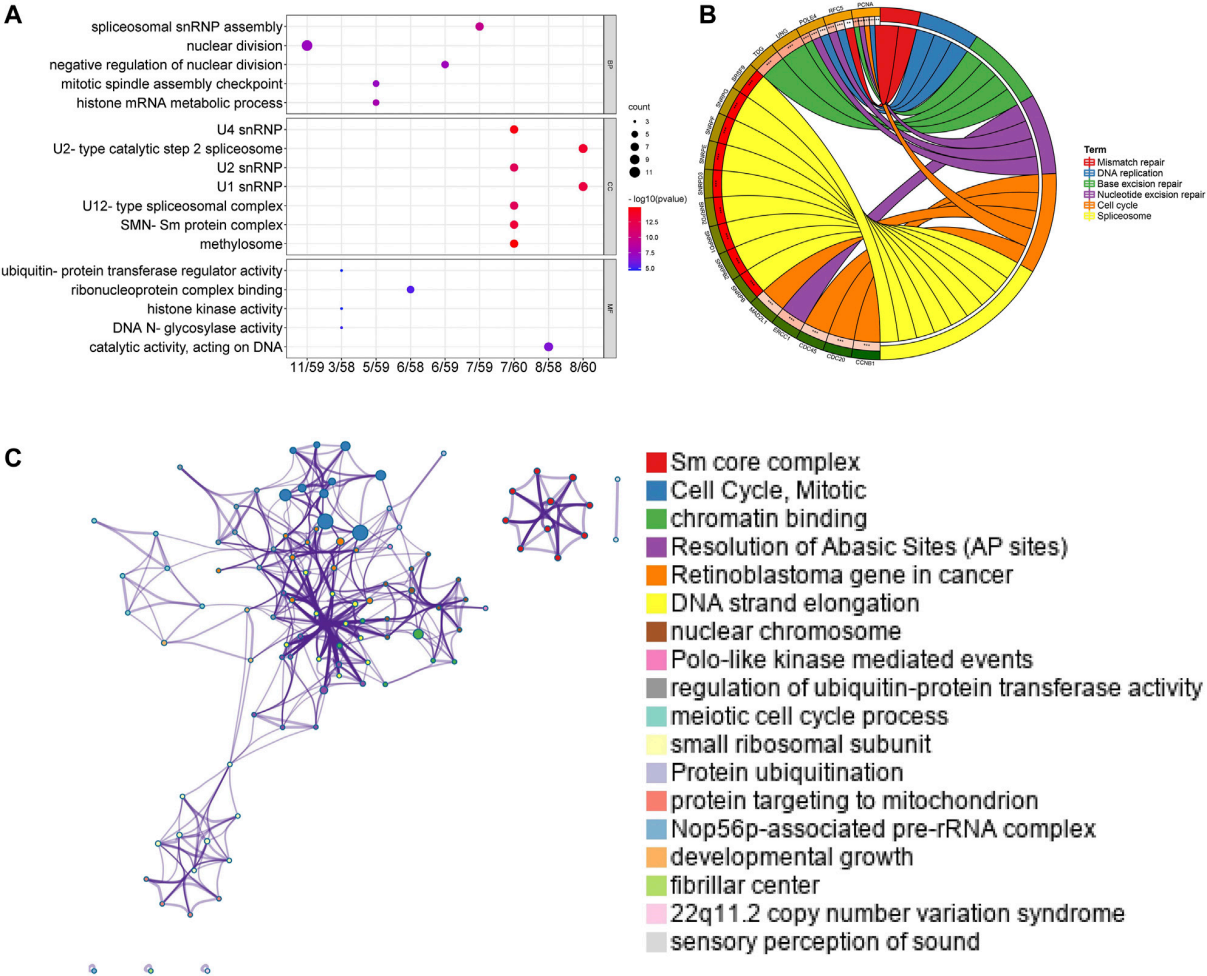
Functional and enrichment analysis for Sm proteins genes and their 50 neighboring genes in LUAD (DAVID 6.8, Metascape). **(A)** GO enrichment analysis of genes for Sm proteins and their 50 neighboring genes (DAVID 6.8). **(B)** Chord diagram of KEGG analyses results. **(C)** Network of seven enriched terms, colored by cluster ID (Metascape).

Afterward, genes for Sm proteins and their 50 neighboring genes were analyzed *via* the tool of DAVID 6.8 for GO and KEGG functional enrichment analysis. The results were shown in Figure 7 and Supplementary Figure S3. Biological processes (BP) included spliceosomal snRNP assembly, histone mRNA metabolic process, negative regulation of nuclear division, nuclear division, and mitotic spindle assembly checkpoint (Figure 7A). Cellular components (CC) suggested that SNRP genes existed mainly in methylosome, U4 snRNP, U2-type catalytic step 2 spliceosome, SMN-Sm protein complex, U1 snRNP, U2 snRNP, and U12-type spliceosomal complex (Figure 7A). Molecular function (MF) indicated that SNRP genes were related to the structural constituents of catalytic activity, acting on DNA, ribonucleoprotein complex binding, DNA N-glycosylase activity, histone kinase activity, and ubiquitin-protein transferase regulator activity (Figure 7A). KEGG pathway enrichment analysis suggested that spliceosome, base excision repair, nucleotide excision repair, cell cycle, dna replication, and mismatch repair were significantly associated with the alterations of sm proteins. The complex process of mRNA/RNA splicing was shown in Supplementary Figure S4. In addition, the KEGG chord plot showed that PCNA was related to 5 vital KEGG pathways. Thus, PCNA may be a very critical gene (Figure 7B). To better understand the association among different enriched terms, the KEGG network of enriched terms, colored by cluster ID, was constructed *via* the online tool of Metascape (Figure 7C).

### Associations Between Genes for Sm Proteins and Immune Infiltration

**FIGURE 8:**
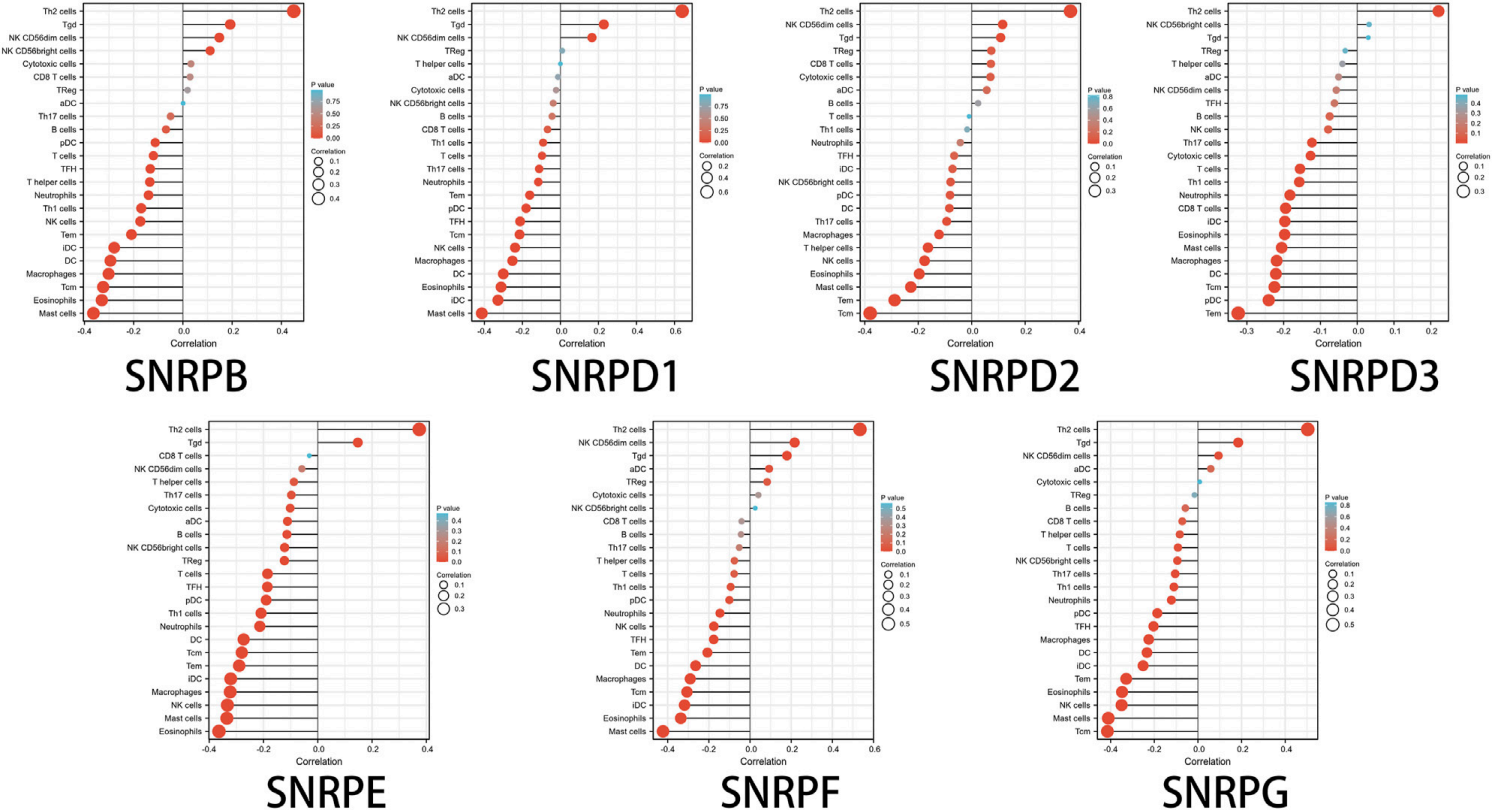
The lollipop charts of the associations between 24 immune cell types and Sm proteins.

High genes for Sm proteins expression was considerably related to abundance infiltration of immune cell in the tumor immune microenvironment (Figure 8). Thus, further research of genes for Sm proteins in 24 immune cell populations manifested that genes for Sm proteins had positive correlations to Th2 cells, Tgd cells, but an adverse correlation to Th1 cells, mast cells, and NK cells.

## Discussion

Lung cancer has been the major cause of tumor occurrence and mortality around the world (Sung et al., 2021). LUAD accounts for half part of lung cancer with an extremely low survival rate (Chen et al., 2016). Although the research on the biological characteristics of lung adenocarcinoma has made some progress in the past few decades, the tumor progression mechanism of lung adenocarcinoma is still unclear. Through literature review and online database bioinformatics analysis and experimental verification, this study verified that genes for Sm proteins are highly expressed in lung adenocarcinoma and have a significant impact on the prognosis of lung cancer. SnRNPs, mainly responsible for splicing the pre-RNA into mRNA, are composed of seven genes for Sm proteins and a small eponymous snRNA (Chari et al., 2008). Accurate splicing is essential to ensure normal cellular function like cell proliferation, apoptosis, migration, and invasion.

This study covered many aspects, including differential expression analysis, association with clinicopathological parameters, survival analysis, gene mutations, co-expression, functional enrichment analysis, and correlation with immune infiltration, revealing the roles of SNRPB/D1/D2/D3/E/F/G in LUAD.

Previous study reported that SNRPB promotes the tumor formation of NSCLC by regulating RAB26 and SNRPB may predict response to cisplatin-based chemotherapy for NSCLC patients (Liu et al., 2019; Liu et al., 2021). Here, the mRNA expression of SNRPB was found to be upregulated in LUAD compared to non-cancer tissues. Also, SNRPB is highly expressed in cervical cancer (Zhu et al., 2020), glioma (Correa et al., 2016), and hepatocellular cancer (Zhan et al., 2020), which is a prognostic factor. The mechanistic study also reported that overexpressed SNRPB played a carcinogenic role in the progression of LUAD and was mediated by c-Myc (Peng et al., 2020). Moreover, SNRPB was highly correlated with TP53 mutation, tumor grades, and cancer stages. Interestingly, our study revealed that SNRPB up-regulation was associated with poor OS in LUAD patients, especially in patients with smoking history. Taken together, our study revealed that SNRPB could promoted tumor progression in LUAD.

It has been generally accepted that SNRPD1 is related to SLE (Riemekasten et al., 1998). However, only a few studies have reported the role of SNRPD1 in human cancers. SNRPD1 down-regulation was related to poorer survival in patients with ovarian cancer (Bao et al., 2020). SNRPD1 up-regulation contributed to breast cancer cell proliferation (Dai et al., 2021). Here, we found that SNRPD1 expression was significantly up-regulated in LUAD compared to normal tissues or normal cell lines. Furthermore, SNRPD1 expression was correlated with TP53 mutation, tumor grades, and cancer stages. Overexpression of SNRPD1 could be relevant to the poor OS in LUAD patients. Multivariate Cox regression analysis indicated that SNRPD1 was an independent prognostic factor for poorer OS of LUAD patients. Taken together, all of the above findings support the opinion that SNRPD1 plays an essential role in the carcinogenic effect of LUAD.

SNRPD2 is found closely associated with several cancers, including triple-negative breast cancer (TNBC) (Koedoot et al., 2021) and hepatocellular carcinoma. Interestingly, SNRPD2 and SNRPG were not only major pathogenic genes of Alzheimer’s disease but also bridge genes (Tao et al., 2020). However, it is not yet clear to us the specific role that SNRPD2 plays in LUAD. Here, we report SNRPD2 was found overexpressed in LUAD. Additionally, high SNRPD2 expression was correlated with TP53 mutation, tumor grades, and cancer stages.

SNRPD3 (smD3), like SNRPD2, also plays a vital role in TNBC. Moreover, several studies have shown that SNRPD3 expression is relevant to breast cancer (Koedoot et al., 2021) and NSCLC (Blijlevens et al., 2020). For instance, it was investigated that silencing SNRPD3 was able to promote TP53 expression and kill NSCLC cells effectively (Olst et al., 2017). In this study, higher SNRPD3 expression was found in LUAD. Moreover, SNRPD3 expression was associated with TP53 mutation, tumor grades, and cancer stages. Multivariate Cox regression analysis suggested a strong association between overexpressed SNRPD3 with the poor OS of LUAD patients, which seemed congruous with the oncogenic role of SNRPD3.

SNRPE protein is a core component of Sm proteins, which has been reported in some malignancies, including bladder cancer (Tapak et al., 2015), prostate cancer (Anchi et al., 2012), hepatocellular carcinoma (Jia et al., 2011), and non-small cell lung cancer (Valles et al., 2012). As an example, SNRPE was a deregulated RNA metabolism-related genes of LUAD by bioinformatics analysis (Valles et al., 2012). Similarly, in our report, higher SNRPE expression was found in LUAD than in normal lung tissues. Furthermore, SNRPE expression was relevant to TP53 mutation and tumor grades. Overexpressed SNRPE was an independent prognostic factor for poorer OS in LUAD patients by cox analysis. Multivariate Cox regression analysis indicated that SNRPE was an independent prognostic factor for poorer OS of LUAD patients, suggesting that SNRPG played a carcinogenic role in LUAD. Taken together, our study revealed that SNRPE could promoted oncogenesis in LUAD.

SNRPF was a novel biomarker of colorectal cancer *via* complex gene interaction networks (Sun et al., 2016). However, the role of SNRPF was rarely reported in LUAD. In our study, highly expressed SNRPF was found in LUAD compared to non-cancer tissues. Moreover, abnormal SNRPF expression could be relevant to TP53 mutation, tumor grades, and cancer stages. Multivariate Cox regression analysis indicated that SNRPDF was an independent prognostic factor for poorer OS of LUAD patients. Given the above results, our study showed that SNRPF could play a carcinogenic role in LUAD.

Downregulated SNRPG could inhabit glioblastoma cells proliferation by p53 signaling pathway (Lan et al., 2020). In this paper, higher mRNA expression of SNRPG was explored in LUAD. Furthermore, SNRPD1 expression could also be relevant to TP53 mutation, tumor grades, and cancer stages. Multivariate Cox regression analysis indicated that SNRPG was an independent prognostic factor for poorer OS of LUAD patients, suggesting that SNRPG played a carcinogenic role in LUAD.

Notably, in this paper, we assessed the relation between genes for Sm proteins and 22 immune infiltration cells in LUAD. genes for Sm proteins gene expressions were positively related to the infiltration of Th2 cells and negatively related to the infiltration of Th1 cells, mast cells, and NK cells. Furthermore, prior studies also suggested that spliceosome was tightly associated with the immune microenvironment. Nevertheless, further studies are still needed to develop immunosuppressants of individual genes for Sm proteins members and apply them to the diagnostic of LUAD.

In this work, there are a few limitations that need to be recognized. Firstly, the data used to assess the prognostic worth of genes for Sm proteins in LUAD patients were mainly from the TGCA database and GEO database. Although the TGCA and GEO sequencing data were experimentally confirmed, additional large sample studies on LUAD patients from other databases are necessary to validate our results. Secondly, Cox analysis indicated that the expressions of SNRPD1//E/F/G were correlation to shorter OS in LUAD. Consequently, SNRPD1/E/F/G were considered as independent prognostic factors and potential prognostic biomarkers for LUAD. Finally, further experiments in cells and animal models are needed to elucidate the underlying mechanisms of how genes for Sm proteins play roles in LUAD.

Taken together, our work illustrates that SNRPD1/E/F/G could independently predict the prognostic outcome of LUAD and was correlated with immune infiltration. Our findings laid the foundation for further exploration on the promising therapy target’s role of Sm proteins in LUAD.

## Data Availability

The original contributions presented in the study are included in the article/Supplementary Material, further inquiries can be directed to the corresponding authors.

## Abbreviations

BP, biological processes; CC, cellular components; FP, free progression; GEO, gene expression omnibus; GO, gene ontology; GTEx, genotype-tissue expression project; KEGG, kyoto encyclopedia of genes and genomes; LUAD, lung adenocarcinoma; MF, molecular function; OS, overall survival; PPS, post-progression survival; SLE, systemic lupus erythematosus; ssGSEA, single-sample gene sets enrichment analysis; snRNA, small nuclear RNA; TCGA, the cancer genome atlas.
