# Supplementary figures and images for: SNRPD1/E/F/G Serve as Potential Prognostic Biomarkers in Lung Adenocarcinoma
Source: Front Genet. 2022 Mar 3;13:813285. doi: 10.3389/fgene.2022.813285 (PMC8959887; doi:10.3389/fgene.2022.813285)

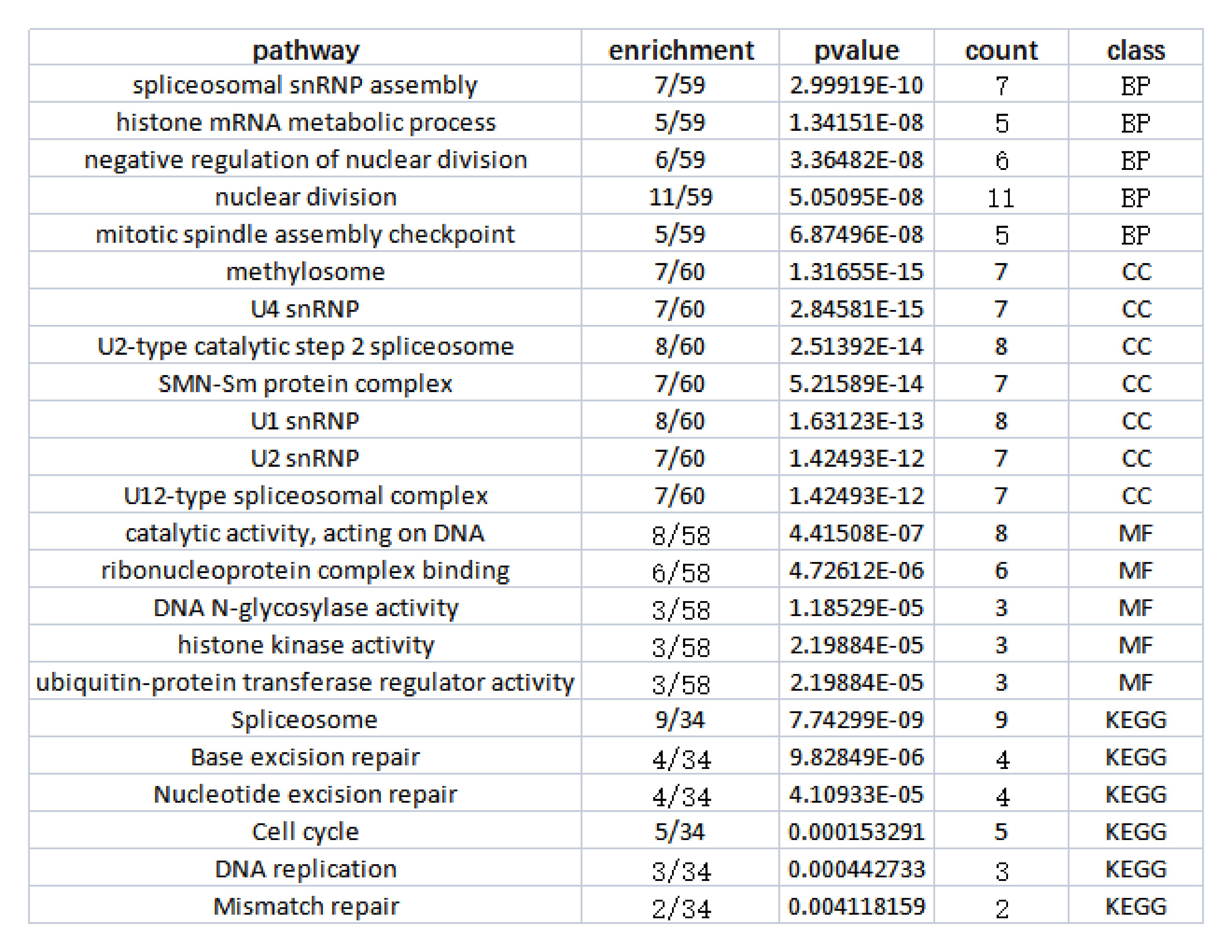

Supplement: Supplementary file 1 [file Image3.JPEG]

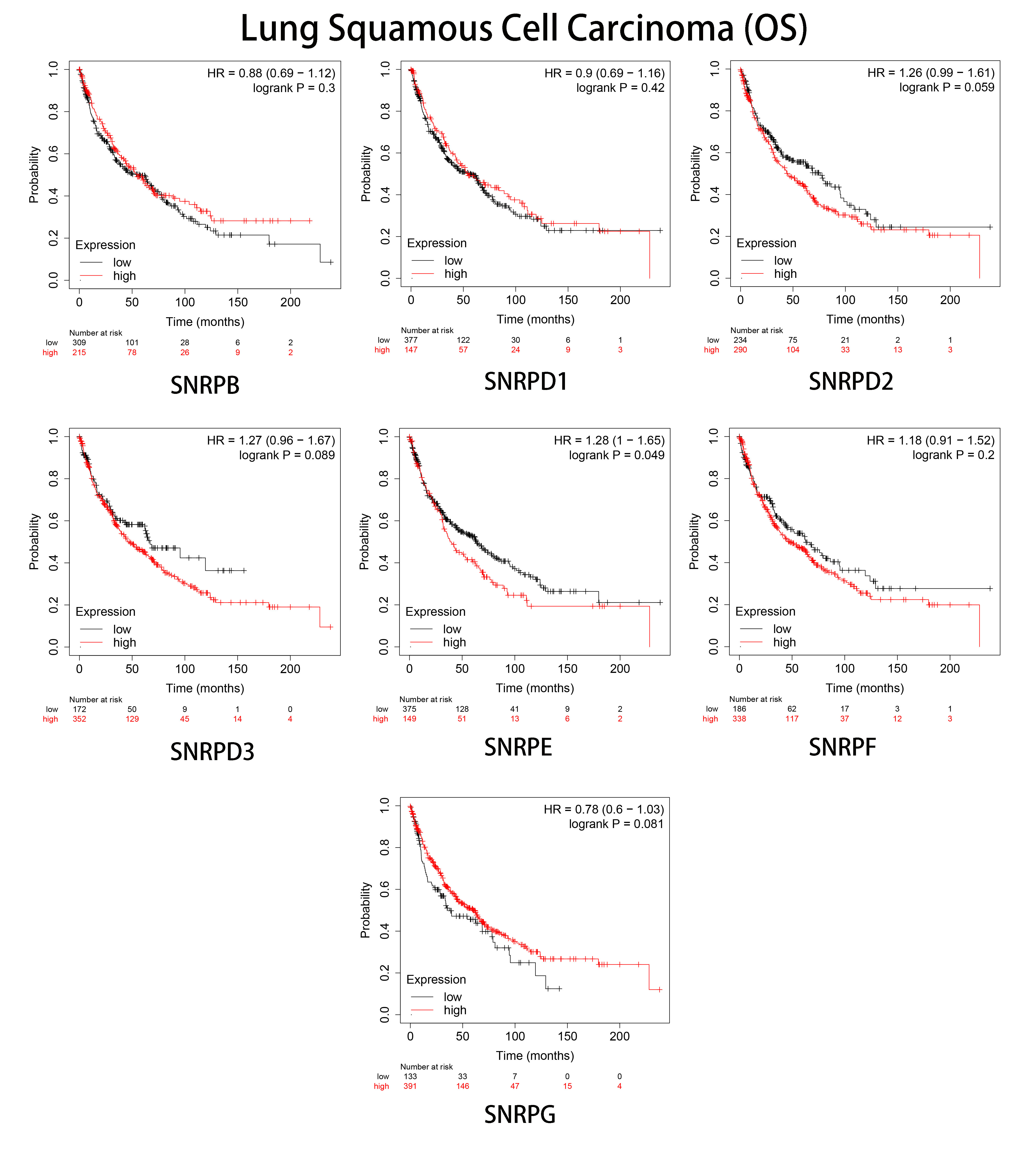

Supplement: Supplementary file 2 [file Image1.JPEG]

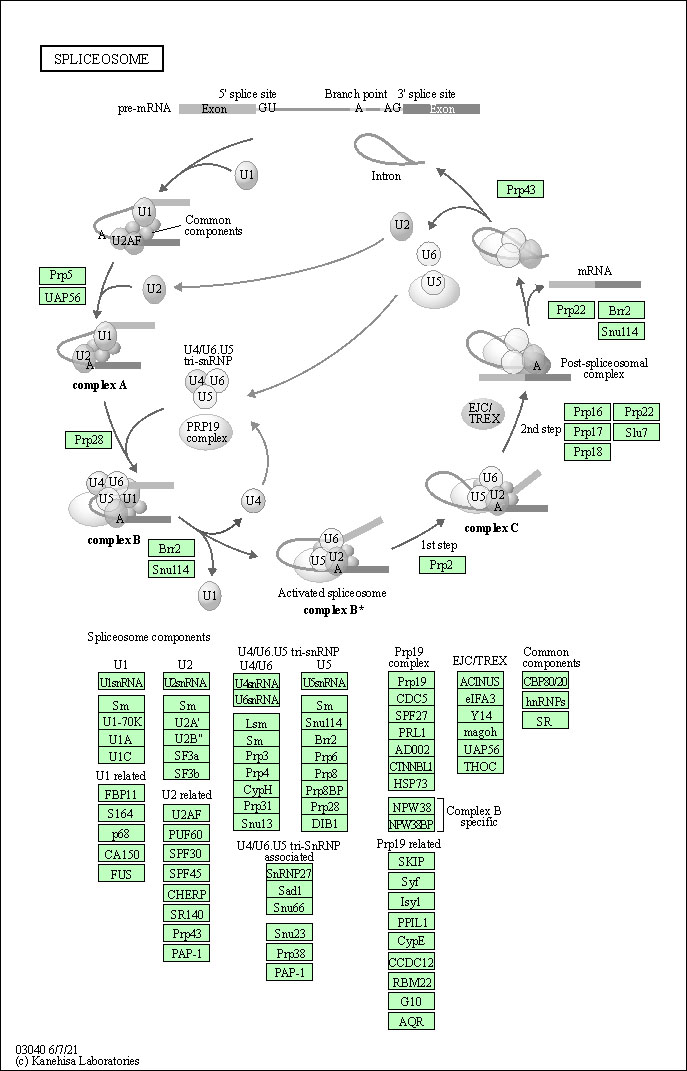

Supplement: Supplementary file 3 [file Image4.JPEG]

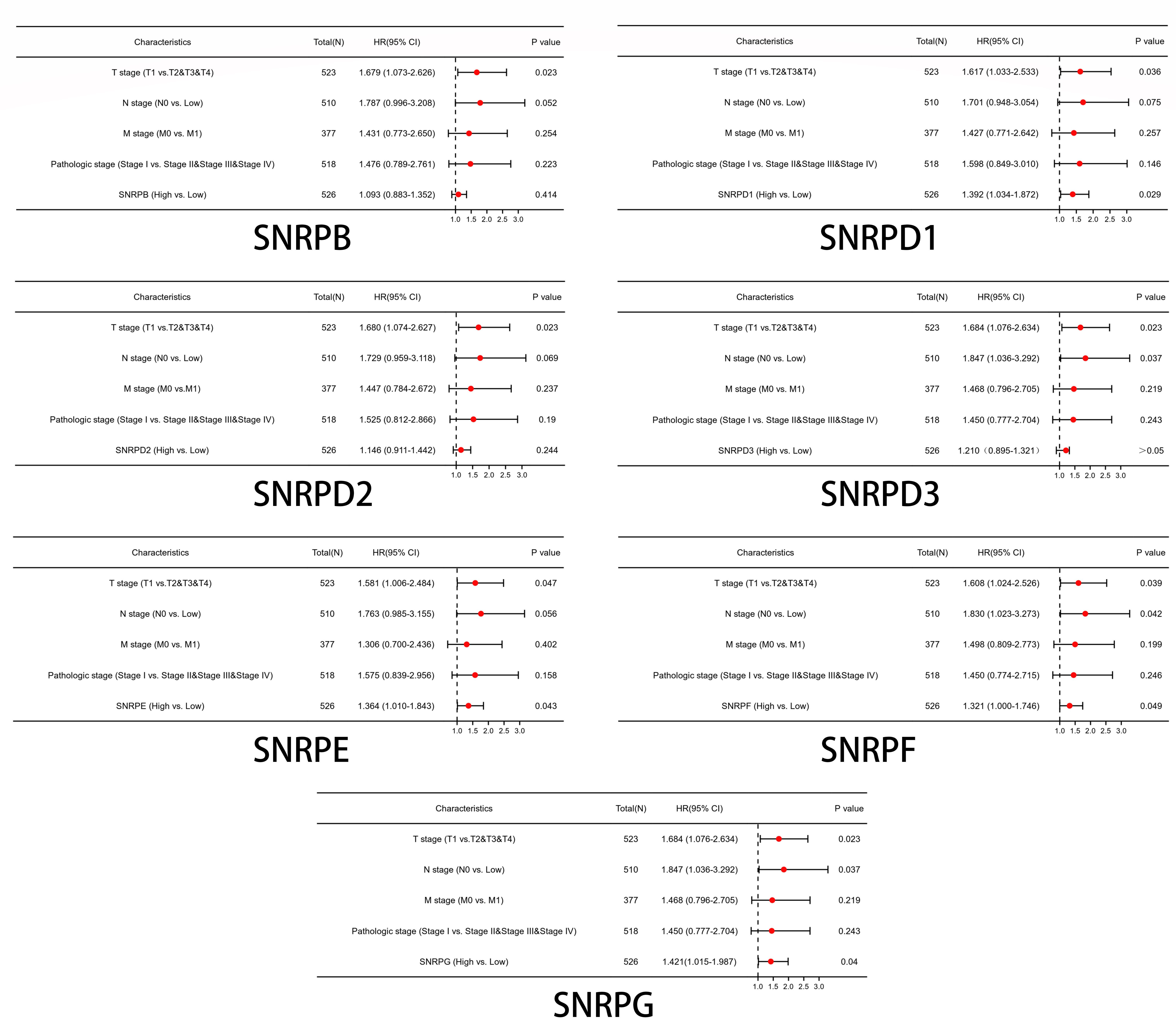

Supplement: Supplementary file 4 [file Image2.JPEG]
