# Supplementary material for: SNRPD1/E/F/G Serve as Potential Prognostic Biomarkers in Lung Adenocarcinoma
Source: Front Genet. 2022 Mar 3;13:813285. doi: 10.3389/fgene.2022.813285 (PMC8959887; doi:10.3389/fgene.2022.813285)
Supplement: Supplementary file 5 [file DataSheet1.docx]

**Supplementary Table 1. Basic characteristics of 535 LUAD patients**

| **Variables** |  | **LUAD patients(n=535)** |
| --- | --- | --- |
| Age, n (%) | <=65 | 255 (49.4%) |
|  | >65 | 261 (50.6%) |
| Gender, n (%) | Female | 286 (53.5%) |
|  | Male | 249 (46.5%) |
| Smoker, n (%) | No | 75 (14.4%) |
|  | Yes | 446 (85.6%) |
| T stage, n (%) | T1 | 175 (32.9%) |
|  | T2 | 289 (54.3%) |
|  | T3 | 49 (9.2%) |
|  | T4 | 19 (3.6%) |
| N stage, n (%) | N0 | 348 (67.1%) |
|  | N1 | 95 (18.3%) |
|  | N2 | 74 (14.3%) |
|  | N3 | 2 (0.4%) |
| M stage, n (%) | M0 | 361 (93.5%) |
|  | M1 | 25 (6.5%) |
| Pathologic stage, n (%) | Stage I | 294 (55.8%) |
|  | Stage II | 123 (23.3%) |
|  | Stage III | 84 (15.9%) |
|  | Stage IV | 26 (4.9%) |
| Primary therapy outcome, n (%) | PD | 71 (15.9%) |
|  | SD | 37 (8.3%) |
|  | PR | 6 (1.3%) |
|  | CR | 332 (74.4%) |
| Residual tumor, n (%) | R0 | 355 (95.4%) |
|  | R1 | 13 (3.5%) |
|  | R2 | 4 (1.1%) |
| Anatomic neoplasm subdivision, n (%) | Left | 205 (39.4%) |
|  | Right | 315 (60.6%) |
| number_pack_years_smoked, n (%) | <40 | 188 (50.9%) |
|  | >=40 | 181 (49.1%) |
| OS event, n (%) | Alive | 343 (64.1%) |
|  | Dead | 192 (35.9%) |
| Age, median (IQR) |  | 66 (59, 72) |

**Supplementary Table 2. Primer sequence information**

| Primer | Primer sequence (5'--3') |
| --- | --- |
| SNRPB-Forward | CTGGTCTCAATGACAGTAGAGGG |
| SNRPB-Reverse | GGGACCCATAGGAGGTCTCATA |
| SNRPD1-Forward | GAATTGAAGAACGGAACACAGGT |
| SNRPD1-Reverse | TCCACAAGTAGTGTATCCAGAGG |
| SNRPD2-Forward | AGTCAAGAACAATACCCAAGTGC |
| SNRPD2-Reverse | ATGTTGCAGTGCCTATCGAAG |
| SNRPD3-Forward | CATTGAAGCAGAGGACAACATGA |
| SNRPD3-Reverse | TCTTTAACATGGGTGCGTTCTT |
| SNRPE-Forward | TGCAGCCCATCAACCTCATC |
| SNRPE-Reverse | GCCTTCTATCCGCATATTCACTT |
| SNRPF-Forward | AGGTCATAGTCCTGTTTGGCG |
| SNRPF-Reverse | GAATGTTGCAGGCTACTCTCTG |
| SNRPG-Forward | AAGACCTCCAAGGAGTGGTAA |
| SNRPG-Reverse | TGGAGAGAGGACATGGGTTT |
| GAPDH-Forward | GCACCGTCAAGGCTGAGAAC |
| GAPDH-Reverse | GGATCTCGCTCCTGGAAGATG |

**Supplementary Table 3. Univariate proportional hazard analysis of snRNPs expressions and OS for LUAD patients**

| **Characteristics** | **Total(N)** | **Univariate analysis** | |
| --- | --- | --- | --- |
|  |  | Hazard ratio (95% CI) | P value |
| Age (<=65 vs. >65) | 516 | 1.223 (0.916-1.635) | 0.172 |
| Gender (Female vs. Male) | 526 | 1.070 (0.803-1.426) | 0.642 |
| Smoker (No vs. Yes) | 512 | 0.894 (0.592-1.348) | 0.591 |
| T stage (T1&T2 vs. T3&T4) | 523 | 1.277 (0.904-1.804) | 0.023* |
| N stage (N0 vs. Low) | 510 | 2.601 (1.944-3.480) | <0.001 |
| M stage (M0 vs. M1) | 377 | 2.136 (1.248-3.653) | 0.006* |
| Pathologic stage (Stage I vs. Stage II& Stage III &Stage IV) | 518 | 2.933 (2.173-3.958) | <0.001* |
| SNRPB (High vs. Low) | 526 | 1.162 (0.973-1.388) | 0.097 |
| SNRPD1 (High vs. Low) | 526 | 1.259 (0.985-1.609) | 0.065 |
| SNRPD2 (High vs. Low) | 526 | 1.240 (1.028-1.497) | 0.025* |
| SNRPD3 (High vs. Low) | 526 | 1.081 (0.815-1.433) | 0.589 |
| SNRPE (High vs. Low) | 526 | 1.389 (1.081-1.785) | 0.010* |
| SNRPF (High vs. Low) | 526 | 1.258 (1.008-1.569) | 0.042* |
| SNRPG (High vs. Low) | 526 | 1.103 (0.908-1.341) | 0.322 |
